# Polyandry in dragon lizards: inbred paternal genotypes sire fewer offspring

**DOI:** 10.1002/ece3.1447

**Published:** 2015-03-24

**Authors:** Celine H Frère, Dani Chandrasoma, Martin J Whiting

**Affiliations:** 1 GeneCology Research Centre, University of the Sunshine CoastMaroochydore DC, Sunshine Coast, Queensland, 4558, Australia; 2 Department of Biological Sciences, Macquarie UniversitySydney, New South Wales, 2109, Australia

**Keywords:** Cryptic female choice, genetic benefits, genotype reconstruction, GERUD, inbreeding, polyandry, reptile, sexual selection, sperm competition

## Abstract

Multiple mating in female animals is something of a paradox because it can either be risky (e.g., higher probability of disease transmission, social costs) or provide substantial fitness benefits (e.g., genetic bet hedging whereby the likelihood of reproductive failure is lowered). The genetic relatedness of parental units, particularly in lizards, has rarely been studied in the wild. Here, we examined levels of multiple paternity in Australia's largest agamid lizard, the eastern water dragon (*Intellagama lesueurii*), and determined whether male reproductive success is best explained by its heterozygosity coefficient or the extent to which it is related to the mother. Female polyandry was the norm: 2/22 clutches (9.2%) were sired by three or more fathers, 17/22 (77.2%) were sired by two fathers, and only 3/22 (13.6%) clutches were sired by one father. Moreover, we reconstructed the paternal genotypes for 18 known mother–offspring clutches and found no evidence that females were favoring less related males or that less related males had higher fitness. However, males with greater heterozygosity sired more offspring. While the postcopulatory mechanisms underlying this pattern are not understood, female water dragons likely represent another example of reproduction through cryptic means (sperm selection/sperm competition) in a lizard, and through which they may ameliorate the effects of male-driven precopulatory sexual selection.

## Introduction

Over the past four decades, experimental and empirical research has generated a large body of evidence for the genetic benefits of polyandry (Baer and Schmid-Hempel 1999; Fisher et al. 2006; Eizaguirre et al. 2007; Klemme et al. 2008; Gowaty et al. 2010). From these studies, we have learnt that when females mate with multiple males, it may increase their fitness (Tregenza and Wedell 1998; Fisher and Lara 1999; Byrne and Whiting 2008) and that of their offspring (Klemme et al. 2008; Firman 2011). An adaptive but largely untested explanation for polyandry is that females minimize inbreeding depression by biasing fertilization toward sperm from genetically compatible males or males with good genes (Tregenza and Wedell 2002; Bretman et al. 2009). But both experimental research and empirical research have shown that not all polyandrous females bias paternity against their relatives following copulation (e.g., lemon shark (*Negaprion brevirostris*), Feldheim et al. 2004; common shrews (*Sorex araneus*), Stockley 1997; and black field crickets, (*Teleogryllus commodus*), Jennions et al. 2004). Avoiding breeding with a close relative may therefore be unnecessary if there is no cost. However, when breeding with close relatives imposes fitness costs (Keller 1998; Baer and Schmid-Hempel 1999; Frère et al. 2010; Walling et al. 2011; Nielsen et al. 2012), females may also be able to minimize inbreeding depression by biasing paternity toward more heterozygous males. This hypothesis requires testing across a broad spectrum of taxa.

Female polyandry in reptiles is particularly common, yet our understanding of its potential benefits remains limited (Uller 2008; Uller and Olsson 2008; Keogh et al. 2013). Typically, females that mate with multiple males stand to gain either direct or indirect benefits, although there is currently no evidence for direct benefits in lizards (Uller and Olsson 2008). Additionally, most reptiles lack parental care and, as such, benefits of female polyandry are believed to have evolved as a mechanism to minimize inbreeding, minimize genetic incompatibility, and maximize the genetic quality and/or diversity of mates (Uller and Olsson 2008). While a few reptilian studies have shown that multiple mating enhances female fitness (Madsen et al. 1992; Olsson et al. 1994; Eizaguirre et al. 2007; Noble et al. 2013), only one study thus far has shown that male siring success correlated with the extent of genetic relatedness to the mother (Olsson et al. 1996). In the European sand lizard, females are promiscuous and mate with males as they encounter them. Because of the obvious risks of mating with a relative, especially given that relatedness may increase with spatial proximity, females are able to discriminate sperm and bias fertilization toward less closely related males (Olsson et al. 1994, 2004).

Evolutionary theory predicts that females should favor males that are more distantly related and therefore either genetically dissimilar or genetically more compatible (e.g., (Slatyer et al. 2012). Unfortunately, very few studies have examined the link between parental relatedness and fitness in polyandrous systems. This is particularly true for lizards: With the exception of the sand lizard (*Lacerta agilis*), we have a remarkably poor understanding of reproductive success as a simple function of genetic relatedness between parents. Here, we investigated the extent of multiple paternity in the eastern water dragon (*Intellagama lesueurii*) and examined whether male siring success correlated with (1) its heterozygosity coefficient and/or (2) its extent of relatedness to the mother.

## Methodology

### Study system

The eastern water dragon is a long-lived, large, semi-aquatic diurnal agamid lizard native to the east coast of Australia (Thompson 1993). They often retreat to water when threatened and either sleep submerged up to their necks or on branches overhanging water (Courtice 1981; Thompson 1993). Males are larger (snout-vent length) than females, have relatively larger heads, and are conspicuously red ventrally, beginning in the neck region and including the limbs (Cuervo and Shine 2007). Eastern water dragons display elaborate mating and social behaviors. Males display alternative mating tactics (ARTs), switching between either aggressively defending a territory or adopting satellite behavior (Baird et al. 2012). They are also highly social and show nonrandom patterns of association within and between sexes that are independent of relatedness (Strickland et al. 2014). The strongest bonds are found between females although males do form strong associations with females and both sexes at times avoid members of the same and opposite sex (Strickland et al. 2014).

### Study site

We studied water dragons in Lane Cove National Park (LNP), which is situated in a bushland valley in northern metropolitan Sydney, Australia. The Lane Cove River courses through LNP and most water dragons occur within close proximity of the shoreline. The vegetation in the park consists mainly of casurina (*Casurina glauca*) woodlands along the riverbanks, isolated patches of Sydney blue gum (*Eucalyptus saligna*) forest, or areas containing a combination of blackbutt (*E. pilularis*), turpentine (*Syncarpia glomulifera*), and blue gum. The riverbank varies from densely vegetated to open, cleared areas consisting of wood chips and logs. We conducted fieldwork along 2 km of river in LNP (beginning: 33°47′29.54″S, 151° 9′20.40″E, end: 33°47′12.84″S, long: 151° 8′53.20″E) from September 2010 to January 2012. Males establish territories along the riverbank, which is typically in close proximity to walking trails or picnic areas; consequently, they were accustomed to humans and allowed relatively close approach (ca. 2–10 m).

### Sample collection

We collected blood samples from the caudal veins of 143 adult eastern water dragons and tail samples (ca. 3–5 mm) from 169 offspring from 22 clutches. (Eastern water dragons have tails that are much longer than the body and are particularly thin in babies. Tail tips are fragile and break naturally as they grow.) Mothers were known for 18 clutches because we brought late-term gravid females into captivity and kept them in outdoor enclosures until they gave birth. The eggs were then incubated in the laboratory in damp river sand within plastic containers and checked daily. Blood sample was stored in 90% ethanol, and tail samples were stored in 75% ethanol. Adults were caught by noosing or by hand.

### Microsatellite analysis

We extracted DNA from tissue and blood samples using QIAGEN DNeasy extraction kit l (Qiagen, Shanghai, China). Adults and offspring genotypes were obtained using nine microsatellite loci developed for eastern water dragons (Frère et al. 2011; : EWD6, 15, 16, 24, 34, 46, 51, 62 and 69). PCR amplification procedures and conditions also followed Frère et al. (2011). All loci were found to be in Hardy–Weinberg equilibrium (HWE), and no linkage disequilibrium was detected. Loci were examined for patterns consistent with null alleles using Microchecker (The University of Hull, Hull, UK) (Frère et al. 2011), and no loci had null alleles.

### Assessing multiple paternity using paternal genotype reconstruction

To test for multiple paternity across our 22 clutches (18 with known mothers and 5 with unknown mothers), we used the program GERUD2.0 (Jones 2005). The exclusion probabilities for all nine microsatellites can be found in Table1. GERUD2.0 determines the minimum number of father combinations to explain a given array of mother–offspring and ranks them by calculating their relative probabilities using patterns of Mendelian segregation and genotype frequencies measured from the population (in our case *n* = 143 adults). Using the most likely minimum-father combination, Gerud2.0 was used to reconstruct the paternal genotypes for the 18 known mother–offspring clutches.

**Table 1:** Measures of genetic diversity of the nine microsatellite loci derived from the 143 adults used in this study. All loci were found to be in Hardy–Weinberg equilibrium, and no linkage disequilibrium was detected

Locus	Number of Alleles	Observed Heterozygosity	Expected Heterozygosity	Exclusion Probability
EWD 34	12	0.826	0.831	0.67
EWD 6	4	0.470	0.461	0.25
EWD 15	6	0.134	0.133	0.06
EWD 16	10	0.772	0.789	0.62
EWD 24	13	0.792	0.847	0.70
EWD 46	4	0.591	0.653	0.40
EWD 51	9	0.805	0.744	0.52
EWD 62	20	0.836	0.841	0.78
EWD 69	15	0.852	0.858	0.72
Over all loci	93	0.675	0.693	0.99

### Statistical analysis

We used the maternal and reconstructed paternal genotypes in COANCESTRY (V1.0.1.1) (Wang 2011) to measure relatedness estimates between the maternal and paternal genotypes using Wang (Wang 2002) and the degree of paternal inbreeding coefficient (*F*) using Ritland (Ritland 1996). Here, the biases introduced when the same markers are used to assign paternity and estimates of parental relatedness and heterozygosity should not apply given that paternal genotypes used in this study were not identified using paternity analyses (Wang 2010) but were reconstructed using the maternal and offspring genotypes as described above. This allowed us to test whether the degree of parental genetic relatedness and paternal inbreeding had an impact on who sired the most offspring within a clutch. Male reproductive success (Rs) was measure by the number of sired offspring divided by clutch size. We investigated the relationship between Rs and the degree of parental genetic relatedness and paternal inbreeding fitting a generalized linear model (GLM) using a binomial distribution. To account for heterogeneity, we weighted the model by clutch size.

In addition, we investigated whether the probability of encounters between close paternal and maternal kin increased with geographic proximity. This is because we hypothesized that if females have high chances of encountering close kin of the opposite sex, then this may influence the evolution of inbreeding avoidance mechanisms. We examined this possibility by investigating whether geographic proximity measured by the distance between captured locations correlated with pairwise relatedness measures calculated as above.

## Results

### Multiple paternities

Paternal genotype reconstruction analysis conducted in GERUD2.0 (exclusion probability over all loci = 0.99, see Table1) allowed us to find strong evidence for multiple paternity in eastern water dragons with 13.6% (3/22) of clutches sired by a minimum of one father, 77.2% (17/22) of clutches sired by a minimum of two fathers, and 9.2% (2/22) of clutches sired by a minimum of three fathers (see Table2 for details).

**Table 2:** Presence of multiple paternity in the 22 clutches of eastern water dragons measured using GERUD 2.0

Family	Known mother	Brood size	Number of fathers	Number of offspring sired by father 1	Number of offspring sired by father 2	Number of offspring sired by father 3
B1_1	Yes	8	2	6	2	
B1_12	Yes	7	3	3	2	2
B1_14	Yes	11	2	9	2	
B1_17	Yes	5	2	3	2	
B1_18	Yes	8	2	6	2	
B1_19	Yes	11	2	9	2	
B1_2	Yes	9	2	7	2	
B1_21	Yes	8	2	5	3	
B1_24	Yes	9	1	9		
B1_25	Yes	10	2	7	3	
B1_26	Yes	13	2	8	5	
B1_27	Yes	13	2	8	5	
B1_4	Yes	7	2	6	1	
B1_5	Yes	9	2	8	1	
B1_8	Yes	8	2	6	2	
B2_15	Yes	8	2	4	4	
B2_17	Yes	4	1	4		
B2_21	Yes	8	1	8		
B1_10	No	8	3	3	2	3
B1_13	No	5	2	3	2	
B1_15	No	5	2	3	2	
B1_20	No	4	2	3	1	

### The relationship between male reproductive success and heterozygosity, partner relatedness, and spatial overlap

**Figure 1:**
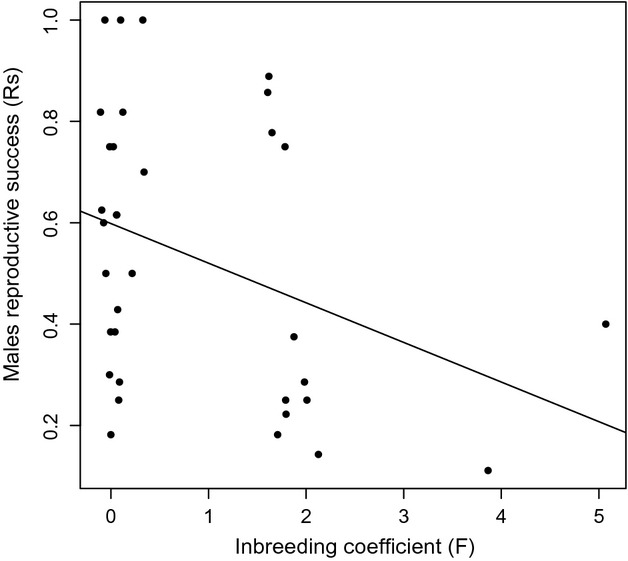
Significant relationship between males' reproductive success (Rs) and their inbreeding coefficient (*F*). Rs was measured by dividing the number of sired offspring by the size of the clutch. Significance was assessed using GLM with binomial distribution. In this analysis, only clutches with known mothers were used (*n* = 18). From these, we used GERUD 2.0 to deduct the paternal genotype. When GERUD 2.0 assigned multiple sires to a clutch, we used the most likely minimum-father combination to assign paternal genotypes to clutch (see Table2 for exclusion probabilities).

**Figure 2:**
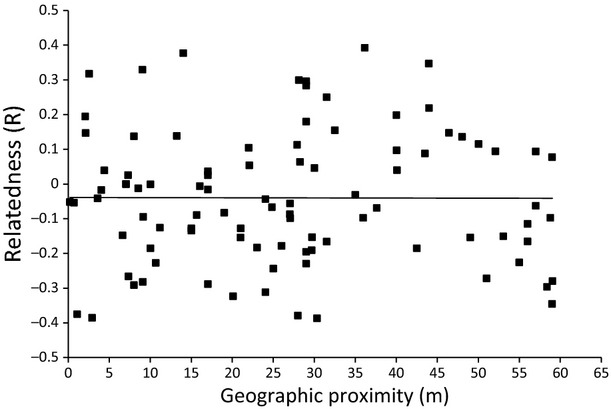
No evidence for a relationship between geographic proximity and relatedness between male and female eastern water dragons at Lane Cove National Park (LNP, females = 64, males = 79).

We found a male's reproductive success (number of sired offspring/clutch size) significantly correlated with their inbreeding coefficient (GLM with binomial distribution: average effect = −0.3402, SE = 0.11, *z*-value = −3.068, *P* = 0.002). The more heterozygous males were, the more the offspring they sired (Fig.1). However, we did not find that males who sired the most offspring within a clutch were more distantly related to the females than males who sired fewer offspring. The extent of genetic similarity between male and female dragons was unrelated to spatial proximity at LNP based on capture locations (Fig.2).

## Discussion

Here, we present evidence that polyandrous female eastern water dragons bias fertilization toward sperm from more heterozygous males rather than less genetically related males. This is consistent with findings from other systems such as splendid fairy-wrens (*Malurus splendens*) (Keith et al. 2005), sand lizards (Olsson et al. 1996), and eastern chipmunks (*Tamias striatus*) (Bergeron et al. 2011). Female mate choice for more heterozygous males has, however, been documented in birds such as the lekking wire-tailed manakin (*Pipra filicauda*) (Ryder et al. 2009) and the cooperatively breeding white-browed sparrow weaver (*Plocepasser mahali*) (Harrison et al. 2013). Our study, however, provides the first evidence for female bias fertilization toward more heterozygous males in lizards.

There may be several reasons why we did not find a correlation between male reproductive success and parental relatedness. First, it may be that mating with close relatives does not incur major fitness costs (e.g., naked mole rat *Heterocephalus glaber*, Reeve et al. 1990) and, as such, avoiding breeding with a close relative may be unnecessary. Second, and in contrast to species with extended kinship (e.g., elephants, Archie et al. 2007), there may be no need for mechanisms to avoid inbreeding because the probability of encountering kin within close geographic proximity is low. While the probability of encountering close relatives within our study population is unknown, we expect it to be lower than species with extended family groups. Indeed, neither geographic proximity (our study) nor social preferences (see Strickland et al. 2014) correlate with relatedness. While further work is required to assess the extent (if any) of inbreeding depression in this species/population (Balloux et al. 2004), mechanisms to avoid inbreeding are only expected to evolve when the cost of tolerating inbreeding exceeds that of avoiding it (Waser et al. 1986).

By favoring more heterozygous males, female eastern water dragons might simply seek to gain maximum genetic benefits from polyandry. Heterozygosity has been, for instance, linked to greater disease resistance (Reid et al. 2007) and increased reproductive success (e.g., Ryder et al. 2009) and as such may be used as a proxy for the genetic quality of mates (e.g., the lekking wire-tailed manakin, Ryder et al. 2009). While heterozygosity has been hypothesized as a plausible umbrella mechanism for the evolution of female polyandry (Rubenstein 2007; Taylor et al. 2014), the benefits of multiple mating in lizards have been difficult to establish (Uller and Olsson 2008; Keogh et al. 2013). First, very few lizard species provide obvious parental care beyond simple parent–offspring associations (While et al. 2014), precluding direct benefits beyond ejaculate products (Uller and Olsson 2008). Indirect benefits such as good genes have likewise been difficult to establish. For example, a series of mating experiments in the promiscuous skink *Eulamprus heatwolei* found no evidence for indirect benefits and the most parsimonious explanation for polyandry was high male encounter rates coupled with low mating costs for females (Keogh et al. 2013), an hypothesis highlighted in a recent review of multiple mating in reptiles (Uller and Olsson 2008). Eastern water dragons can occur at high densities, and mate encounter rates are high. For example, at Roma Street Parkland, there is a population in excess of 580 animals and females may overlap with up to 42 males (Gardiner et al. 2014). However, multiple matings as a consequence of frequent encounters may not be the only model that best explains water dragon mating dynamics because males control space and, as a result, females may be able to avoid particular males by closely associating with the dominant male. Furthermore, this model implies a low costs to reproduction which is likely not the case given the biased paternity we detected. Nevertheless, females may still be susceptible to coercion and ultimately, copulations that they may not favor. The success of males with higher levels of heterozygosity may be a result of two possible postcopulatory mechanisms of sexual selection. First, females may be capable of sperm selection and cryptically favor the sperm of more heterozygous males. Second, the sperm of more heterozygous males may fare better during sperm competition if sperm competition correlates with particular male genotypes that are also less heterozygous. This pattern is different to sand lizards, in which females favored less related males and this could be due to the differing probabilities of encountering kin in the two systems (high in sand lizards, low in water dragons). While the exact mechanisms driving fertilization success remains elusive, our results are consistent with an indirect benefits model of sexual selection via multiple mating.

In summary, Eastern water dragons experience intense precopulatory sexual selection in the form of male–male combat (Baird et al. 2012) and sexual coercion (pers. obs.). While sexual coercion by male water dragons may limit a female's precopulatory mate choice, we provide evidence that postcopulatory mechanisms of sexual selection are rife and may help level the sexual conflict playing field. While we cannot be sure that the mechanism is sperm selection per se, as opposed to sperm competition, the outcome is the same and is likely to be adaptive although this requires future testing.
